# Rabies knowledge and prevention practices in Gombe state, Nigeria: a community-based comparative cross-sectional study of rabies hotspot and non-hotspot areas

**DOI:** 10.1186/s12889-025-21309-2

**Published:** 2025-01-16

**Authors:** Eugene Chidi Eugene, Pius Odunze, Bile Nuhu, Olukemi Titilope Olugbade, Mishel Dauda, Abiodun Egwuenu, Olugbemisola W. Samuel, Hilary I. Okagbue

**Affiliations:** 1 Sydani Institute for Research and Innovation, Sydani Group, Abuja, Nigeria; 2 Department of Public Health, Gombe State Ministry of Health, Gombe, Gombe State, Nigeria; 3 Department of Community Medicine, College of Medical Sciences, Gombe State University, Tudun Wada, Gombe State, Nigeria; 4 Department of Medical Services, Ministry of Defence2 Division Nigeria Army, Ibadan, Oyo State, Nigeria; 5 Department of Community Health, Obafemi Awolowo University Teaching Hospital Complex, Ile-Ife, Osun State Nigeria; 6 Ministry of Agriculture, Gombe, Gombe State, Nigeria; 7 Charite Universitatmedizin/Humboldt University, Berlin, Germany

**Keywords:** Dogs, Public Health, Rabies, Nigeria, Vaccination

## Abstract

**Background:**

Rabies remains a significant public health concern in Nigeria, particularly in rural areas with limited awareness and resources. Gombe State is recognized as a rabies hotspot, facing challenges in controlling the spread of the disease. This study aimed to assess and compare the knowledge and prevention practices related to rabies among community members in hotspot and non-hotspot areas of Gombe State.

**Methods:**

A community-based comparative cross-sectional study was conducted in Gombe State. Multistage sampling technique was used to select 816 eligible respondents from selected households with pet dogs or cats (408 each from hotspot and non-hotspot areas). A semi-structured, pre-tested digital interviewer-administered questionnaire was used to collect data on socio-demographics, rabies knowledge, and prevention practices. Data were analyzed using SPSS version 26. The univariate and bivariate analysis compared rabies knowledge and prevention practices at *p* value < 0.05. Logistic regression identified predictors of good practice.

**Results:**

Respondents from hotspot areas demonstrated better overall knowledge of rabies compared to those from non-hotspot areas. However, a higher proportion of respondents from non-hotspot areas exhibited better rabies prevention practices, particularly in terms of vaccinating their dogs within the previous year. Factors such as older age (AOR: 0.47, 95% CI: 0.27– 0.84), farming occupation (AOR: 0.48, 95% CI: 0.27 – 0.84), and good overall rabies knowledge (AOR: 3.04, 95% CI: 1.37 – 6.73) were significant predictors of rabies prevention practices in the hotspot area.

**Conclusion:**

Targeted educational interventions and tailored prevention strategies are needed to improve rabies awareness and practices, especially among specific demographic groups like older individuals and farmers. Enhancing overall knowledge of rabies and promoting consistent vaccination practices for pets are crucial steps towards reducing the incidence of rabies in both hotspot and non-hotspot areas.

**Supplementary Information:**

The online version contains supplementary material available at 10.1186/s12889-025-21309-2.

## Background

Rabies, a fatal viral disease affecting the central nervous system of mammals, including humans, remains a significant public health concern in many parts of the world, particularly in low-income, rural areas of Africa and Asia [1]. The disease is caused by the rabies virus of the Rhabdoviridae family, which is primarily transmitted through the saliva of infected animals, typically via a bite or scratch. While rabies is most associated with wild animals such as raccoons, bats, skunks, and foxes, domestic animals like dogs, cats, and cattle can also be carriers of the virus [1, 2].

The progression of rabies is rapid and devastating [3]. Once the virus enters the body, it travels to the brain and spinal cord, causing inflammation and severe damage to the nervous system. Early symptoms of rabies may include fever, headache, muscle weakness, and numbness around the bite area. As the disease advances, it leads to agitation, confusion, hallucinations, hydrophobia (fear of water), and paralysis, and ultimately results in coma and death. Unfortunately, once clinical symptoms manifest, rabies is irreversible, almost always fatal, and currently no effective treatment for the disease exist [4].

Rabies is classified as one of the neglected tropical diseases (NTDs) by the World Health Organization (WHO) due to its prevalence in underserved communities with limited access to healthcare and veterinary services [5]. Africa and Asia bear the highest burden of human rabies cases, with over 95% of deaths occurring in these regions, and more than 99% of those deaths are attributed to dog bites. Nigeria, being endemic for rabies, faces significant challenges in controlling the spread of the disease, particularly in rural areas with limited awareness and resources [6, 7].

Globally, there were 14,075.51 incident rabies cases in 2019 and 13,743.44 deaths (95% uncertainty interval: 6019.13–17,938.53), with adolescents and adults under 50 years old making up the bulk of rabies cases [8]. The condition remains a significant public health concern in Nigeria, burdening the country with an estimated 2,500 to 5,000 human deaths annually [9, 10]. Dogs serve as the primary reservoir and transmitter of rabies to humans, responsible for approximately 95% of human rabies cases in the nation [11]. The majority of victims are children under the age of 15 years, reflecting the vulnerability of certain populations to rabies exposure [12]. Apart from the human toll, rabies also affects livestock and domestic animals, posing challenges to agricultural productivity and livelihoods.

Gombe State, situated in Nigeria, is particularly affected and recognized as a hotspot for rabies cases [13]. The region's persisting rabies problem can be attributed to the lack of widespread awareness, limited access to healthcare facilities, and insufficient preventive measures. The incidence of human rabies cases remains alarmingly high, with a significant number of fatalities reported annually. Additionally, the prevalence of stray and unvaccinated dogs in both urban and rural areas of the country further heighten the risk of rabies transmission.

In the hotspot communities of Gombe State, inadequate knowledge and limited implementation of control measures contribute to a high risk of rabies transmission from animals to humans. This situation poses a significant public health threat as community members may not recognize early symptoms, fail to seek timely medical attention after animal exposure, and neglect vaccinating their pets.

Addressing this pressing issue requires immediate intervention to enhance rabies awareness, strengthen preventive measures, and foster collaboration among stakeholders. By conducting a comprehensive community-based comparative cross-sectional study focusing on rabies hotspot and non-hotspot areas, we can assess and compare the level of rabies knowledge and preventive measures among communities residing in these regions. The findings will inform targeted interventions to improve rabies control and prevention efforts in Gombe State, ultimately working towards reducing the burden of rabies in Nigeria and protecting the well-being of its communities.

Gombe State, Nigeria, faces a substantial burden of rabies, especially in rural communities with limited access to healthcare and veterinary services. Despite being preventable through vaccination, rabies remains a serious public health threat, evident from reported cases of dog bites and fatalities in recent years [14]. From 2020 to the present, Gombe State reported 308 cases of dog bites, with 42 confirmed as rabid bites, tragically resulting in four human deaths [15].

To address these challenges, understanding the factors influencing rabies prevalence and transmission is crucial. Identifying knowledge gaps and awareness of rabies prevention, particularly in hotspots, can aid in developing effective control strategies. This community-based comparative cross-sectional study aims to investigate and compare rabies knowledge and prevention measures between hotspot and non-hotspot areas in Gombe State. The study aims to provide valuable insights for targeted interventions and awareness campaigns, empowering communities to protect themselves and their animals effectively against rabies.

Rabies, with its near 100% fatality rate, demands preventive measures through education, vaccination, and proper animal management. Enhancing rabies awareness among community members can lead to early medical intervention after animal bites, increasing the chance of survival. Effective control measures, such as regular pet vaccinations and stray animal management, can create a buffer zone against rabies transmission. Strengthening collaboration among stakeholders, including community members, veterinarians, healthcare providers, and local authorities, is vital for a comprehensive approach to combating rabies effectively.

The study aimed to determine the factors associated with rabies prevention measures among community members in rabies hotspots and non-hotspot areas of Gombe State. The specific objectives are to:

1. Assess and compare the knowledge of community members about rabies in hotspots and non-hotspot areas of Gombe State.
2. Assess and compare the rabies prevention practices of community members in rabies hotspots and non-hotspot areas of Gombe State.
3. Determine the factors associated with good prevention practices among community members in rabies hotspots and non-hotspot areas of Gombe State.

## Methods

### Study area

This study was conducted in Gombe State, which is in the Northeastern geopolitical zone of Nigeria and has a total land area of 20,265sq.km. It lies between latitude 9″30’ and 12″30’N, longitude 8″5’and 11″45’E [16]. It shares boundaries with Adamawa and Taraba State to the South, Bauchi State to the West, Borno State to the East, and Yobe State to the North. Gombe State. It's 2024 projected population, at a growth rate of 3.3%, was 4,090,783, with a population density of about 300 people per square kilometre [17, 18]. With regards to topography, the state is mainly mountainous, undulating and hills in the South-East and flat open plains in the Central, North, North-East, West and North-Western parts [16].

Gombe State is comprised of 11 Local Government Areas (LGAs), with five LGAs identified as rabies hotspots: Akko, Balanga, Billiri, Dukku, and Gombe. These areas have a higher incidence of human rabies cases based on the State’s epidemiological data [9, 19]. In contrast, the non-hotspot LGAs, including Funakaye, Kaltungo, Nafada, Shongom, and Yamaltu Deba, have lower reported cases or no cases of rabies as shown by a scoping review that spanned over a decade, corroborated by epidemiological data [9]. The northern and central senatorial zones each comprise three Local Government Areas (LGAs), whereas the southern senatorial zone includes four LGAs. In terms of hotspot classification, the northern senatorial zone contains one hotspot LGA and two non-hotspot LGAs. The central senatorial zone is characterized by two hotspot LGAs and one non-hotspot LGA. Conversely, the southern senatorial zone has two hotspot LGAs alongside two non-hotspot LGAs.

The state is multi-ethnic in nature, with the main tribes being Awak, Bolewa, Cham, Dadiya, Fulani, Hausa, Jara, Jukun, Kanuri, Kamo, Lunguda, Pero, Tangale, Tera, Tula and Waja. The State’s official language is English; however, Hausa is the commonest local language spoken.

**Fig. 1:**
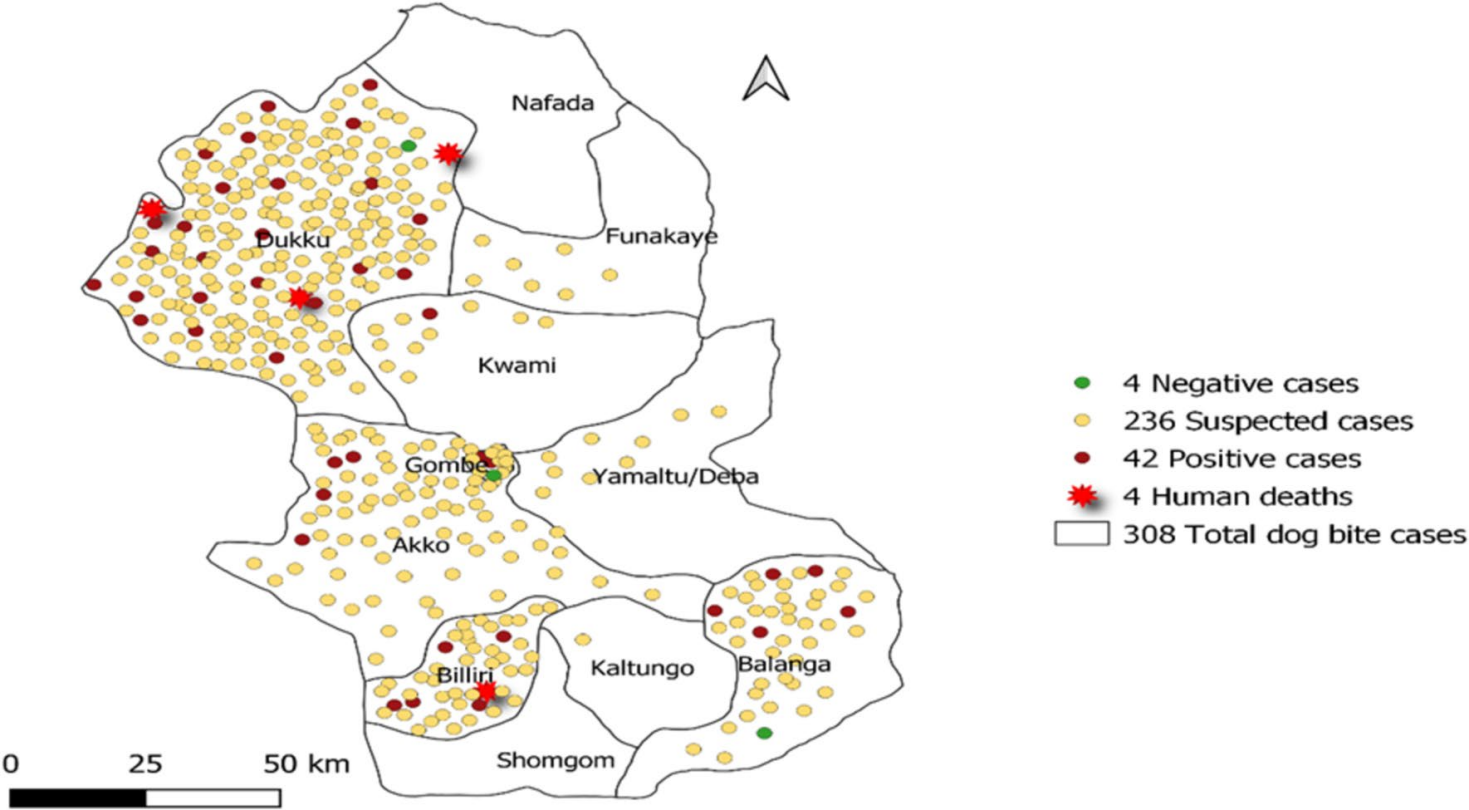
Spot map of the distribution of rabid dog bites, Confirmed dog rabies cases and human deaths in Gombe State. 2020 – 2022

The state has a predominantly rural population with limited access to healthcare services, particularly in remote areas [18]. Socio-economic factors, such as poverty and lack of education, contribute to the vulnerability of local communities to rabies [18]. The rabies surveillance system in Gombe State, Nigeria, involves collaboration between the human health and veterinary sectors for risk assessment and management of potential rabies exposures. Although this collaboration is in place, the system encounters some obstacles, such as limited financial resources, infrastructure deficiencies, restricted communication from higher authorities, and gaps in knowledge regarding rabies prevention and management among healthcare professionals and the public [18]. Spot map showing the distribution of rabid dog bites, confirmed rabies cases and deaths in Gombe State. 2020 – 2022 is shown in Fig. 1.

### Study design

A cross-sectional study design was employed in this study.

### Study population

The study population in the rabies hotspots included members of communities in three hotspot LGAs (i.e., LGAs with a history of confirmed rabid dog bites between 2020 to 2022) and three other LGAs where no confirmed rabid dog bite was reported from 2020 to 2022.

### Inclusion criteria

Any consenting adult (aged ≥ 18 years) who has resided in any of the selected communities for at least one year by the time the study commences and owns a dog and/or cat.

### Exclusion criteria

Eligible community members who were unable to participate in the study due to illness were excluded from consideration.

### Sample size

The minimum sample size, (n), was obtained using the formula for testing for any difference in outcome between two groups [20].

$$\text{n}=\text{DEFF x }{\left(Z\alpha +Z\beta \right)}^{2}\times \text{PQ }{\left(p1-p2\right)}^{2}$$

DEFF (Design effect) = measure of variability during the process of sampling to select survey subjects by any method other than simple random sampling. Taken as 3 in this study.

Z_α_ = standard normal deviate corresponding to 5% level of significance = 1.96.

Z_1_-_β_ = statistical power of the test at 80% = 0.84.

P = Proportion of units to be measured i.e., “good rabies prevention practices.”

Q = Complementary probability of p which is q = 1- p.

p1 = Proportion of respondents with good rabies prevention practices in a rabies hotspot area in previous study – 0.14.

p2 = Proportion of respondents with good rabies prevention practices in a rabies non-hotspot area (assumed to be a 10% [i.e., 0.1] difference from that of the rabies hotspot area) – 0.24.

P = Pooled proportion of the outcome of interest = (p1 + p2)/2 = (0.14 + 0.24)/2 = 0.19.

Q = 1–0.19 = 0.81.

PQ = 0.19 × 0.81 = 0.1539.

(p1 – p2)2 = d = expected difference between the two groups = p1—p2 = (0.14–0.24)^2^ = 0.01.

Substituting these values into the above formula, the calculated sample size was found to be:

$$n=\frac{3*{\left(1.96 + 0.84\right)}^{2} 0.19* 0.81}{{\left(0.14-0.24\right)}^{2}}$$

n = 361.97.

There are three LGAs in each study arm, therefore the sample size was divided into three, i.e., 361.97 ÷ 3 = 120.66 = 121 per LGA.

Assuming a non-response rate of 10%, the adjusted sample size (n’) became.

n’ = n/ 1 – f.

Where f is the correction factor of 0.1

n’ = 361.97/0.9

n’ = 402.2

The adjusted minimum sample size, therefore, was 402 per study arm; because of the three LGAs in each study arm, the sample size was divided into three, i.e., 402.2 ÷ 3 = 134.1 = 134 per LGA.

### Sampling technique

A multistage sampling technique was used in this study.

## Stage one: selection of LGAs

Using computer-generated random numbers, two LGAs were selected per senatorial zone—one hotspot and one non-hotspot LGA. For zones where there is only one LGA in a particular category (e.g., only one hotspot in Gombe North – Dukku, or one non-hotspot in Gombe Central – Yamaltu Deba), those LGAs were automatically selected. In zones where more than one LGA fell into a category, simple random sampling was employed using computer-generated random numbers to ensure unbiased selection. Consequently, the selected rabies hotspot LGAs included Dukku, Akko, and Billiri while the non-hotspot LGAs included Nafada, Yamaltu Deba, and Shongom.

*Stage two: Selection of communitie*s.

A master list spreadsheet containing all LGAs, wards and communities in Gombe State was obtained from the World Health Organization (WHO) in Gombe State and used to select the wards and communities. In Gombe State, three LGAs have 11 wards each, namely: Akko, Dukku, and Gombe. Yamaltu Deba has 12 wards while the remaining LGAs have 10 wards. In all the selected LGAs, 10 wards were selected using simple random sampling by computer-generated random numbers (making a total of 60 wards) and in each selected ward three communities with the largest population were selected (making a total of at least 180 communities). For LGAs with 10 wards, all the wards were automatically selected.

### Stage three: Selection of households and study respondents

In each selected community the approximate geographic centre of the settlement or cluster was located. From the centre, one of the four cardinal directions (East, West, North, or South) was randomly selected by balloting. The data collection started in the first household with a dog in the selected direction. A household was defined as a group of people living together under the same roof and sharing common cooking and eating arrangements.

Following the initial visit, the second and subsequent visits were in dog-owning households closest to the preceding house visited. This was repeated until the appropriate sample size was achieved in that community. The closest dog-owning household was defined as the one that can be reached in the smallest amount of time on foot from the one that was just visited. In each dog-owning household, the owner of the dog or someone with information about the dog was interviewed.

In each selected LGA, a total of 136 dog owners were interviewed across the 10 selected wards. In each ward, across the three selected communities, a total of at most 14 respondents were interviewed. If the required number of respondents was not gotten in a community or ward, then the immediate next community or ward was visited to make up the number.

### Data collection tool

A semi-structured, pre-tested digital interviewer-administered questionnaire with three sections was adapted and used in this study. Sections A, B, and C contain socio-demographics, knowledge of rabies, and rabies control practices respectively. We carried out a pilot test to evaluate the tool's reliability in this setting. Both the knowledge and the preventative practice assessment sections' Cronbach's alpha were determined using the pilot's responses. The knowledge assessment had a Cronbach's alpha of 0.85, which indicates strong internal consistency, while the prevention practice assessment had an alpha of 0.78, which indicates adequate internal consistency.

For the knowledge section, the correct response to 50% of the quantitative metrics and above was considered good knowledge. For the practice section, it was all or none (100%), as failure to engage in even one prevention practice still exposes someone to rabies infection. The 50% cutoff for knowledge level classification was justified on the premise that, since highly knowledgeable people on all facets of the topic are unlikely to be found in a community-based study, a fair understanding of half of the important rabies metrics is deemed sufficient for the community being studied [21]. This is a sensible strategy because prior research has shown that, even in rabies-affected areas, there is variation in community awareness and knowledge of the disease [22]. The questionnaire is attached as a Supplementary material.

### Data collection technique

Sixty research assistants were trained over 3 days across the 6 LGAs. The digital interviewer-administered questionnaire in the Kobo Collect Android application was used to collect data from eligible respondents in the field.

To ensure the validity and reliability of our research, we conducted a pretest of the study tool, involving 40 participants from a Gombe LGA in the State. This pretest was crucial in assessing the questionnaire's clarity, relevance, and cultural appropriateness. Both research assistants and respondents provided valuable feedback on the questionnaire. Minor adjustments were made to improve clarity and flow, ensuring a smooth and efficient data collection process.

### Data handling and processing

The data collected was downloaded from the Kobo Toolbox website as an Excel spreadsheet and exported to the Statistical Product and Services Solutions (SPSS) version 25 software where it was processed and analysed.

### Data analysis

The data was analysed based on the specific objectives of the study.

Firstly, univariate analysis of the socio-demographic characteristics of the respondents was carried out and the basic descriptive statistics was presented in frequencies, percentages, mean and standard deviation or median and interquartile range where appropriate. The methods of data analysis for the objectives are outlined.

#### Objective 1

To achieve Objective 1, univariate analysis was conducted to assess the level of knowledge of rabies among community members in selected rabies hotspots and non-hotspot areas of Gombe State. Subsequently, bivariate analysis (chi-square test) was employed to compare the knowledge levels between the two areas. A p-value of < 0.05 was considered statistically significant.

#### Objective 2

Univariate analysis was performed to assess the rabies prevention practices of the community members in the rabies hotspot and non-hotspot areas. Subsequently, bivariate analysis (i.e., chi-square) was carried out to compare the rabies control practices in the study areas. A p-value of < 0.05 was considered statistically significant.

#### Objective 3

Bivariate (i.e., chi-square) and multivariable (i.e., binary logistic regression) analytical methods were used to determine the factors associated with good control practices among the community members.

## Results

### Findings

A total of 816 eligible respondents were enrolled in the study, with 136 per LGA. All the selected respondents consented to participate in the study, giving a 100% response rate. The mean age was 39.1 ± 13.0. The mean age of females was 38.7 ± 12.0, while that of males was 39.3 ± 13.2.

Socio-demographic Characteristics of Hotspot and Non-Hotspot Respondents in Gombe state is presented in Tables 1 and 2 respectively.

**Table 1:** Socio-demographic characteristics of Hotspot and Non-Hotspot Respondents

**Characteristic**	**Rabies Hotspot** **(*n* = 408)** **Frequency (%)**	**Non-Hotspot** **(n = 408)** **Frequency (%)**	$$\chi$$^**2**^	**df**	**p-value**
**Age group (years)** < 20 20 – 24 25 – 29 30 – 34 35 – 39 40 – 44 45 – 49 50 – 54 55 – 59 60 – 64 65 – 69 70 – 74 75 – 79 ≥ 80	7(1.7) 18(4.4) 66(16.2) 56(13.7) 59(14.5) 44(10.8) 47(11.5) 43(10.5) 17(4.2) 26(6.4) 11(2.7) 9(2.2) 1(0.2) 4(1.0)	6(1.5) 36(8.8) 38(9.3) 154(37.7) 39(9.6) 42(10.3) 29(7.1) 26(6.4) 9(2.2) 10(2.5) 10(2.5) 6(1.5) 0(0) 3(0.7)	83.292	13	< 0.0001*
**Mean/Median age**	Median: 39 (IQR: 30,50)	Mean: 37.5 ± 11.9			
**Sex** Female Male	51(12.5) 357(87.5)	128(31.4) 280(68.6)	42.431	1	< 0.0001*
**Marital status** Married Single Divorced Separated Widow(er)	344(84.3) 57(14.0) 0(0.0) 2(0.5) 5(1.2)	302(74.0) 88(21.6) 6(1.5) 4(1.0) 8(2.0)	19.108	4	**0.001 **^**$**^
**Highest level of education** None Koranic only Primary Secondary Tertiary	93(22.8) 71(17.4) 80(19.6) 108(26.5) 56(13.7)	94(23.0) 65(15.9) 72(17.6) 121(29.7) 56(13.7)	1.429	4	0.839

**Table 2:** Socio-demographic characteristics of Hotspot and Non-Hotspot Respondents continued

**Occupation** Artisan Civil Servan Driver Farmer Hunter Trader Others^#^	14(3.4) 52(12.7) 14(3.4) 229(56.1) 51(12.5) 31(7.6) 17(4.2)	14(3.4) 34(8.3) 16(3.9) 233(57.1) 25(6.1) 49(12.0) 37(9.1)	**24.288**	**3**	< 0.0001*
**Religion** Christianity Islam Others	154(37.7) 252(61.8) 2(0.5)	149(36.5) 257(63.0) 2(0.5)	0.132	2	0.936^$^
**Ethnic group** Bolewa Fulani Hausa Kanuri Tangale Tera Others^b^	9(2.2) 141(34.6) 56(13.7) 27(6.6) 134(32.8) 2(0.5) 39(9.6)	34(8.3) 106(26.0) 65(15.9) 1(0.2) 73(17.9) 23(5.6) 106(26.0)	110.881	1	< 0.0001*
**Monthly income** < N30,000 N30,000 – N59,000 ≥ N60,000	210(51.5) 129(31.6) 69(16.9)	255(62.5) 112(27.5) 41(10.0)	12.681	2	0.002*

^*^ = statistically significant; ^$^ = Likelihood ratio; Others^#^ = Religious leaders, Students, Unemployed, Herbalist, Retiree; Others^b^ = Cham, Dadiya, Igbo, Jara, Jukun, Lungudu, Pero, Tula,Wuja, Yoruba, Angas, Awak, Baganji, Kushi, Wurukun

Both groups of respondents were similar regarding highest level of education and religion. They however differed based on age (*p* < 0.0001), sex (*p*** < **0.0001), marital status (*p* = 0.001), occupation (*p*** < **0.0001), ethnic group (*p*** < **0.0001), and monthly income (*p* = 0.002). While the age distribution of respondents in non-hotspot were normally distributed, that of those from hotspot area were not. Majority of the respondents were of the Hausa/Fulani ethnic group, with a combined 197(48.3%) of them from the hotspot area and 171 (41.9%) from the non-hotspot area.

### Knowledge towards rabies in hotspots and non-hotspot areas

Knowledge of rabies among study respondents in rabies hotspot and non-hotspot areas in Gombe State is presented in Table 3, while the overall level of knowledge of rabies among the study respondents in rabies hotspot and non-hotspot areas is presented in Table 4.

**Table 3:** Knowledge of rabies among study respondents in rabies hotspot and non-hotspot areas in Gombe State

**Knowledge variables**	**Rabies Hotspot** **(*n* = 408)** **Frequency (%)**	**Non-Hotspot** **(*n* = 408)** **Frequency (%)**	$\chi$^**2**^	**df**	**p-value**
**Ever heard of rabies** **Yes** **No**	310(76.0) 98(24.0)	246(60.3) 162(39.7)	23.121	1	< 0.0001*
**Causes of Rabies** Correct response Wrong response	255(62.5) 153(37.5)	155(38.0) 253(62.0)	49.021	1	< 0.0001*
**Animals that transmit Rabies** Correct response Wrong response	291(71.3) 117(28.7)	218(53.4) 190(46.6)	27.828	1	< 0.0001*
**Transmission of rabies** Correct response Wrong response	287(70.3) 121(29.7)	181(44.4) 227(55.6)	56.296	1	< 0.0001*
**Symptoms of rabies** Correct response Wrong response	192(47.1) 216(52.9)	163(40.0) 245(60.0)	4.193	1	0.041*
**Treatability after symptoms and signs** Correct response Wrong response	85(20.8) 323(79.2)	94(23.0) 314(77.0)	0.580	1	0.446
**Prevention of rabies** Correct response Wrong response	294(72.1) 114(27.9)	203(49.8) 205(50.2)	42.621	1	< 0.0001*
**Ideal frequency of dog vaccination** Correct response Wrong response	261(64.0) 147(36.0)	167(40.9) 241(59.1)	43.418	1	< 0.0001*
**Where vaccine can be sourced** Correct response Wrong response	259(63.5) 149(36.5)	118(30.0) 275(70.0)	89.929	1	< 0.0001*
**Source of rabies info** Right source Wrong source	309(75.7) 99(24.3)	245(60.0) 163(40.0)	23.027	1	< 0.0001*

^*^statistically significant

**Table 4:** Overall level of knowledge of rabies among the study respondents in rabies hotspot and non-hotspot areas in Gombe State

**Overall knowledge variable**	**Rabies Hotspot** **(*n* = 408)** **Frequency (%)**	**Non-Hotspot** **(*n* = 408)** **Frequency (%)**	$\chi$^**2**^	**df**	***p*-value**
**Rabies knowledge level** Good Poor	309(75.7) 99(24.3)	233(57.1) 175(42.9)	31.737	1	< 0.0001*
**Median knowledge score**	8.0 (IQR: 6.0,9.0)	6.0 (IQR: 0.0,8.0)			

^*^statistically significant

The groups differed significantly based on all the rabies knowledge assessment items except for the item “treatability of rabies after development of signs and symptoms” (*p* = 0.446).

The median overall knowledge score was 7 (IQR: 0.0,8.0). While 309 (75.7%) participants from the hotspot area had overall good knowledge of rabies, only 233 (57.1%) of non-hotspot participants had good overall knowledge.

### Rabies prevention practices in rabies hotspot and non-hotspot areas

Rabies prevention practices among study respondents in rabies hotspot and non-hotspot areas of Gombe State is presented in Table 5 which shows a significant difference in rabies prevention practices between the two groups. In the hotspot area, 299 respondents (73.3%) had vaccinated their dogs within the previous year. In contrast, a higher proportion of respondents (82.4%, or 336 individuals) from the non-hotspot area had vaccinated their dogs during the same timeframe.

**Table 5:** Rabies prevention practices among study respondents in rabies hotspot and non-hotspot areas of Gombe State

**Variable**	**Rabies Hotspot** **(*n* = 408)** **Frequency (%)**	**Non-Hotspot** **(*n* = 408)** **Frequency (%)**	$\chi$^**2**^	**df**	***p*-value**
**Dogs/Cats confined to the house** Yes No	165(40.4) 243(59.6)	112(27.5) 296(72.5)	15.352	1	< 0.0001*
**Dogs vaccinated in the last 1 year** Yes No	299(73.3) 109(26.7)	336(82.4) 72(17.6)	9.719	1	0.002*

^*^statistically significant

### Other prevention practices

The comparative analysis of other rabies-related experiences and practices revealed some notable differences between the hotspots and the non-hotspot area. However, no confirmatory tests were conducted and were not tabulated. When asked if they had encountered a rabid animal in their community, 199 (48.8%) of respondents in the hotspot area answered affirmatively, while 207 (50.7%) in the non-hotspot area reported the same. Regarding dogs diagnosed with rabies, 66 (16.2%) of dog owners in the hotspot area acknowledged such diagnoses, with 19 (28.8%) of them responding appropriately by seeking post-exposure prophylaxis and euthanizing the infected animal. In contrast, 14.5% of dog owners in the non-hotspot area reported diagnoses of rabies, with 40.6% of them taking the correct actions.

For cats, 35 (8.6%) owners in the hotspot area stated their cats had been diagnosed with rabies, but only two (0.06%) responded appropriately. In comparison, 19 (4.7%) of cat owners in the non-hotspot area reported diagnoses of rabies, with six (31%) of them reacting correctly to diagnoses. In terms of dog bites or scratches, 68 (16.7%) of individuals in the hotspot area had experienced such incidents, with 15 (22.1%) responding correctly. In the non-hotspot area, 46 (11.3%) had been bitten or scratched by a dog, and 11 (24.0%) of them exhibited the right response.

Regarding cat bites or scratches, 45 (11.0%) of individuals in the hotspot area reported such incidents, with 8 (17.8%) responding appropriately. In contrast, 15 (3.7%) of individuals in the non-hotspot area experienced cat bites or scratches, but none took the correct actions afterward.

Lastly, the overall rabies prevention practices among study respondents in rabies hotspot and non-hotspot areas of Gombe State is presented in Table 6.

**Table 6:** Overall Rabies prevention practices among study respondents in rabies hotspot and non-hotspot areas of Gombe State

**Variable**	**Rabies Hotspot** **(*n* = 408)** **Frequency (%)**	**Non-Hotspot** **(*n* = 408)** **Frequency (%)**	$\chi$^**2**^	**df**	***p*-value**
**Overall rabies prevention practices** Good Poor	66(16.2) 342(83.8)	22(5.4) 386(94.6)	24.659	1	< 0.0001*

^*^statistically significant

Table 6 revealed a significant difference in rabies prevention practices between the two groups. In the hotspot area, only 66 respondents (16.2%) had good rabies prevention practices, compared to 22 respondents (5.4%) in the non-hotspot area. The majority of respondents in the hotspot area (83.8%, 342 individuals) had poor rabies prevention practices, while an even higher proportion (94.6%, 386 individuals) in the non-hotspot area exhibited poor practices.

### Factors associated with prevention practices

Factors associated with prevention practices among community members in rabies hotspots and non-hotspot areas of Gombe State are presented in Table 7. In hotspot areas, significant associations were found for the following factors: age group (*p* = 0.009), occupation (*p* = 0.007), and overall rabies knowledge (*p* = 0.012). In contrast, the bivariate analysis for non-hotspot areas identified different significant associations. Here, occupation remained an associated factor (*p* = 0.003), but it was now along with the level of education (*p* = 0.040).

**Table 7:** Factors associated with prevention practices among community members in rabies hotspots and non-hotspot areas of Gombe State

**Characteristic**	**Rabies Hotspot**		$$\chi$$^**2**^	***p*-value**	**Non**	**Hotspot**	$$\chi$$^**2**^	***p*-value**
	**Good** **Practice f (%)**	**Poor Practice f (%)**			**Good Practice** **f (%)**	**Poor Practice** **f (%)**		
**Age group (years)** Older (≥ 40) Younger (< 40)	23(11.4) 43(20.9)	179(88.6) 163(79.1)	6.771	0.009*	7(5.2) 15(5.5)	128(94.8) 258(94.5)	0.017	0.896
**Sex** Female Male	11(21.6) 55(15.4)	40(78.4) 302(84.6)	1.250	0.264	8(6.3) 14(5.0)	120(93.8) 266(95.0)	0.269	0.604
**Marital status** Married Others	55(16.0) 11(17.2)	289(84.0) 53(82.8)	0.057	0.811	15(5.0) 7(6.6)	287(95.0) 99(93.4)	0.412	0.521
**Occupation** Civil servant Farmer Hunter Trader Others	14(26.9) 25(10.9) 14(27.5) 5(16.1) 8(17.8)	38(73.1) 204(89.1) 37(72.5) 26(83.9) 37(82.2)	13.967	0.007*	5(14.7) 6(2.6) 1(4.0) 1(2.0) 9(13.4)	29(85.3) 227(97.4) 24(96.0) 48(98.0) 58(86.6)	16.104	**0.003**^$^*
**Level of education** Any civil education No civil education	46(18.9) 20(12.2)	198(81.1) 144(87.8)	3.206	0.073	18(7.2) 4(2.5)	231(92.8) 155(97.5)	4.225	0.040*
**Ethnic group** Hausa/Fulani Others	28(14.2) 38(18.0)	169(85.5) 173(82.0)	1.083	0.298	10(5.8) 12(5.1)	161(94.2) 225(94.9)	0.120	0.729
**Monthly income** < N30,000 ≥ N30,000	30(14.3) 36(18.2)	180(85.7) 162(81.8)	1.141	0.285	10(3.9) 12(7.8)	245(96.1) 141(92.2)	2.883	0.090
**Religion** Islam Others	39(15.5) 27(17.3)	213(84.5) 129(82.7)	0.238	0.625	12(4.7) 10(6.6)	245(95.3) 141(93.4)	0.711	0.399
**Overall rabies knowledge** Good Poor	58(18.8) 8(8.1)	251(81.2) 91(91.9)	6.318	0.012*	12(5.2) 10(5.7)	221(94.8) 165(94.3)	0.062	0.803

^*^statistically significant, $ = likelihood ratio

### Predictors of good rabies prevention practices

Lastly, the predictors of good rabies prevention practices in hotspot and non-hotspot areas of Gombe State are presented in Table 8.

**Table 8:** Predictors of good rabies prevention practices in hotspot and non-hotspot areas of Gombe State

Characteristics	AOR	95% CI	*p*-value
**Rabies Hotspot** Age group (years) Older (≥ 40) Younger (< 40) **Occupation** Farmer Others + **Overall rabies knowledge** Good Poor	0.47 1 0.48 1 3.04 1	(0.27– 0.84) (0.27 – 0.84) (1.37 – 6.73)	0.01* 0.01* 0.006*
**Non-Hotspot** **Occupation** Farmer Others + **Level of education** Any civil education No civil education	0.30 1 2.38 1	(0.12 – 0.80) (0.78 – 7.31)	0.016* 0.129

*AOR* Adjusted Odds Ratio, *C.I* Confidence Interval

^*^Statistically significant, Others + = civil servants, artisans, drivers, traders, hunters

Multivariable logistic regression for hotspot area found the age group, occupation and overall rabies knowledge to be statistically significant at *p* < 0.05. Older hotspot participants (AOR: 0.47, 95% CI: 0.27– 0.84) were 53% less likely to have good rabies prevention practice compared with the younger ones. Farmers from the hotpot area were 52% less likely to have good rabies prevention practice (AOR: 0.48, 95% CI: 0.27 – 0.84) compared with other categories of workers. Likewise, those with good overall rabies knowledge among the hotspot respondents had three times higher odds of good rabies prevention practices (AOR: 3.04, 95% CI: 1.37 – 6.73) compared with those with poor overall rabies knowledge.

For the non-hot spot respondents, only occupation was found to be significant at p < 0.05. Farmers from the non-hotspot area were 70% less likely to have good rabies prevention practices compared with non-farmers (AOR: 0.30, 95% CI: 0.78 – 7.31).

## Discussion

This study assessed the knowledge and factors influencing rabies prevention practices in Gombe State, comparing hotspot and non-hotspot areas. The socio-demographic characteristics of the two groups were largely similar in terms of religion and the highest level of education but differed significantly in other aspects. Notably, the hotspot area had a higher proportion of older, married male respondents, and was also home to a larger number of hunters.

The similarity in religion and the highest level of education between the two groups suggests that these factors may not play a significant role in differentiating the knowledge and practices related to rabies prevention. However, the notable differences in the proportion of older, married male respondents and the presence of a larger number of hunters in the hotspot area indicate that specific demographic characteristics and occupational activities may be associated with varying levels of awareness and implementation of rabies prevention measures. The higher proportion of older, married male respondents in the hotspot area may imply that this demographic group could be more exposed to potential rabies risk factors due to their age, marital status, and potentially higher engagement in outdoor activities. Additionally, the larger number of hunters in the hotspot area suggests that individuals with this occupation may have an increased likelihood of encountering rabid animals, thus emphasizing the importance of tailored education and prevention strategies for this specific occupational group. This deduction is relatable to another study finding that revealed hunting as a risk factor and constraint to rabies control [23].

The study showed that a significantly higher proportion of respondents in the hotspot areas—more than three-quarters—had heard of rabies compared to those in non-hotspot areas, with less than two-thirds. The respondents in the hotspot areas demonstrated a significantly higher level of understanding regarding the causes of rabies, animals that transmit rabies, transmission of rabies, and the symptoms of rabies compared to those in non-hotspot areas. These factors contributed to a higher overall level of knowledge about rabies among respondents in the hotspot areas. This higher knowledge level could be due to the prevalence and geographic aggregation of rabies exposures in the hotspot area. It could also be due to differences in the implementation of rabies awareness campaigns, access to healthcare resources, and variations in educational interventions between the high burden and low burden areas. With the high burden of rabies comes increased concern and raised awareness and knowledge of rabies [24]. This was consistent with the finding from a study that showed that residents in rabies hotspot areas had higher knowledge of rabies compared to those in non-hotspot areas [24]. This highlights a potential correlation between the prevalence of rabies and public knowledge of the disease.

The findings on disparity in knowledge have significant implications for public health interventions and policy formulation. Addressing the knowledge gap in non-hotspot areas is crucial for comprehensive prevention and control and to sustain any gains made from the control measures for rabies in the hotspot areas. This could involve targeted educational programmes, community engagement initiatives, and the implementation of comprehensive vaccination campaigns for both humans and animals. By bridging the knowledge disparity, it is possible to enhance the overall understanding of rabies, leading to improved prevention strategies and better health outcomes for communities [25, 26].

The assessed practices included determining the vaccination status of dogs and cats over the past year and assessing the restrictions on animal movements, specifically whether they were allowed to roam beyond the household premises. The itemized rabies prevention practices showed a statistically significant difference between the hotspot and non-hotspot areas, with the hotspot area having a higher proportion of respondents with good individual preventive practices. This could be due to the increased awareness of rabies among communities with a high burden of rabies [24]. This finding was in keeping with that of a study conducted in some rabies hotspots that revealed that a high percentage of pet owners vaccinated their animals against rabies and kept the vaccination records [27].

Collectively, in both hotspot and non-hotspot areas, the majority exhibited inadequate rabies prevention practices, with less than one-fifth of hotspot respondents and approximately one-in-twenty non-hotspot respondents demonstrating good overall practices, and the difference was statistically significant. These findings align with a study conducted in Kwara State, Nigeria, which reported overall poor rabies prevention practices with a low dog vaccination coverage of 30% and a notable prevalence of free-roaming dogs [28]. The observed differences in overall rabies prevention practices could be influenced by various factors, including variations in public awareness and education about the importance of dog vaccination [29].

In the hotspot group, age was found to be significantly associated with rabies preventive practices, showing that nearly twice as many younger individuals exhibited good preventive practices compared to their older counterparts. The low prevalence of rabies and the resulting lack of emphasis on its importance may explain the similar age distribution of preventive practices in the non-hotspot area, as well as the lack of association between age and preventive practices. The significant association between age and rabies preventive practices as seen in the hotpot area is in tandem with other documented study findings [26, 30]. These findings collectively demonstrate the influence of age on rabies preventive practices, highlighting the need for targeted educational and preventive interventions, particularly among specific age groups, to effectively mitigate the risk of rabies transmission.

The study found significant associations between occupation and rabies preventive practices in both hotspot and non-hotspot areas. Although majority of respondents from all occupational groups had poor rabies preventive practices (more than 70%), farmers and traders had the lowest proportion of individuals with good preventive practices in both hotspot and non-hotspot regions. Interestingly, the proportion of hunters from the hotspot area exhibiting good rabies preventive practices was higher compared to the other occupational groups in the same area. This finding may be attributed to increased high risk targeted awareness and sensitization efforts on rabies prevention and control in the high-burden hotspot region, as rabies is a potential occupational hazard for hunters [31]. The associations between occupation and preventive practices have implications for public health interventions aimed at improving rabies prevention. Tailored educational programs, occupational health initiatives, and targeted outreach efforts may be necessary to address the specific needs and challenges associated with different occupational groups, ultimately contributing to more effective rabies control and prevention strategies.

In the hotspot area, multivariable logistic regression highlighted age group, occupation, and overall rabies knowledge as predictors of poor prevention practices. Older individuals in the hotspot area were notably less likely to exhibit good rabies prevention practices compared to younger participants. This was in consonance with another study finding that showed individuals in younger age group were almost thrice more likely to have good prevention practices towards rabies than those in older age group [26]. The lower likelihood of older individuals in a rabies hotspot area to exhibit good prevention practices compared to younger participants can be attributed to various factors. Older individuals may have entrenched beliefs or habits that are harder to change, leading to lower compliance with preventive measures. Additionally, older individuals might have less exposure to updated health information or may perceive themselves as less susceptible to rabies, impacting their preventive behaviours [32].

Farmers in the hotspot region were also significantly less likely to engage in effective prevention practices. Conversely, individuals with good overall rabies knowledge in the hotspot had three times higher odds of practicing good prevention measures compared to those with poor knowledge. This was similar to a study finding that showed good knowledge category as a predictor for good rabies preventive practice [26].

In the non-hotspot area, farmers were less likely to have good rabies prevention practices compared to individuals in other occupations. While the analysis revealed some association between level of education and rabies preventive practices, this however was not found to significantly predict rabies preventive practice. Enhancing overall rabies knowledge through educational campaigns and tailored outreach programs could significantly impact preventive behaviours in both hotspot and non-hotspot areas. The study's results emphasize the need for comprehensive public health strategies that address specific demographic groups to effectively combat rabies transmission.

Focusing on individuals from both hotspot and non-hotspot areas is a core strength of the research. Hence, the study accounts for variations in exposure, awareness, and risk, offering comprehensive insights. However, some limitations are noted. Firstly, funding constraints led to the study being geographically restricted to Gombe State, Northeast Nigeria, and consequently, the findings may not be generalizable to other geopolitical regions in Nigeria or countries with different rabies prevalence and resource settings. The use of self-reported data could lead to recall bias. The potential for misclassification or underreporting cannot be ruled out entirely and is acknowledged as a limitation of this study. We recommend strengthening surveillance systems, improving awareness campaigns, and periodically reassessing hotspot and non-hotspot categorizations to ensure more accurate identification and reporting of rabies cases. Lastly, the categorization of occupations into farmers and "others" was done to address small sample sizes in certain subgroups, ensuring statistical stability in the regression analysis. However, this may have masked associations specific to less-represented occupations, such as hunters and traders. Future studies with even larger sample sizes could explore these occupational groups in greater detail.

## Conclusion

This study conducted on rabies knowledge and prevention practices among respondents in hotspot and non-hotspot areas of Gombe State revealed significant differences between the two groups. Participants from the hotspot area demonstrated better overall knowledge of rabies compared to those from the non-hotspot area. Nevertheless, majority from both hotspot and non-hotspot areas had poor inclusive rabies prevention practices. Factors such as age, occupation, and overall rabies knowledge were found to be predictors of rabies prevention practices in the hotspot area. Older participants and farmers were less likely to have good prevention practices, while those with good overall rabies knowledge were more likely to engage in effective prevention measures. In contrast, in the non-hotspot area, only occupation, specifically being a farmer, was significantly associated with poorer prevention practices. To further strengthen rabies prevention efforts, additional research is needed to explore the underlying reasons for the observed disparities in knowledge and practices between hotspot and non-hotspot areas. This research could inform the development of more effective and targeted interventions.

## Supplementary Information

Supplementary Material 1.

## Data Availability

The data that support the findings of this study will be provided upon reasonable request. However, the questionnaire is provided as Supplementary Material.

## Abbreviations

AOR: Adjusted Odds Ratio
LGA: Local Government Area
LR: Logistic Regression
NTD: Neglected Tropical Diseases
SPSS: Statistical Product and Services Solutions
WHO: World Health Organization
