# Supplementary material for: Rabies knowledge and prevention practices in Gombe state, Nigeria: a community-based comparative cross-sectional study of rabies hotspot and non-hotspot areas
Source: BMC Public Health. 2025 Jan 16;25:177. doi: 10.1186/s12889-025-21309-2 (PMC11737207; doi:10.1186/s12889-025-21309-2)
Supplement: Supplementary file 1 — Supplementary Material 1. [file 12889_2025_21309_MOESM1_ESM.docx]

**Questionnaire on Rabies knowledge and prevention practices in Gombe State: A community based comparative study of rabies hotspot and non-hotspot areas**

**Demographics**

1. Age as at last birthday
2. Sex (1) Male (2) Female
3. Occupation

(1) Butcher (2) Civil Servant (3) Driver (4) Drycleaner (5) Farmer (6) Health care worker (7) Hunter/trader of game meat (8) Miner (9) Private school teacher (10) Religious leader (11) Student (12) Tailor (13) Trader/Artisan (14) Traditional/spiritual healer (15) Transporter (16) Unemployed (17) Other, specify _________________

1. Highest level of education

(1). None (2). Koranic only (3). Primary (4). Secondary (5). Tertiary

1. Marital status (1) Single (2) Married (3) Separated (4) Divorced (5) Widow(er)
2. Monthly income _________
3. LGA _________
4. Community ___________
5. Ward __________
6. Rabies hotspot (1) Yes (2) No
7. Religion (1) Christianity (2) Islam (3) Other, (specify)___________
8. Tribe

(1) Bolewa (2) Cham (3) Dadiya (4) Fulani (5) Hausa (6) Jara (7) Jukun (8) Kanuri (9) Lunguda (10) Pero (11) Tangale (12) Tera (13) Tula (14) Waja (15) Igbo (16) Yoruba

(17) Other, specify _______

**Knowledge of Rabies**

1. Have you ever heard of rabies?

(1) Yes (2) No

If yes,

1. What causes rabies? *Kwayan cutan haukan kare (Hausa language)*

(1) Virus (2) Germ (3) bacteria (4) Witchcraft (5) Starvation and thirst (6) Other______ (7) I don’t know

1. How is rabies contracted?

(1) Bite of animals (2) Saliva (3) Infected animal licking somebody’s wound (4) Infected animal licking somebody’s Intact skin licking (5) Eating meat of animals infected with rabies (6) Sex (7) Inhalation of saliva or dust (8) Others ________

1. What types of animals can transmit rabies disease to humans?

(1) Domestic dogs (2) Stray dogs (3) Bats (4) Cats (5) Others_______

1. What are the symptoms of rabies in dogs or human beings?

(1) Aggressiveness (2) Protruding of the tongue (3) Profuse salivation (4) Dropping of tail (5) Dropping of head and neck (6) Eating of abnormal items (7) Hydrophobia (8) Difﬁculty in swallowing (9) Change in sound (10) Photophobia (11) Others___ (12) I don’t know

1. Can rabies be treated after development of clinical signs and symptoms?
   1. Yes (2) No (3) I don’t know
2. How can rabies be prevented?
3. Vaccination of domestic dogs (2) Vaccination of persons before been bitten (3) (2) Vaccination of persons after been bitten by a rabid animal (4) Prevent domestic dogs from roaming in public and interacting with wild animals (5) Thoroughly cleaning animal bites or scratches with soap and water (5) Killing all suspected rapid dogs (6) Others________
4. How often or how regularly should a dog be vaccinated?

(1) Every year (2) Every two years (3) Every three years (4) Others____

1. Where can the rabies vaccine be obtained from in Gombe State?

_________

1. What is your main source of information about rabies?
   1. Friends or neighbours (2) The media (3) Veterinarians (4) Others _______

**Rabies Prevention Practices**

1. Number of dogs owned____
2. Duration of dog ownership (years)_______
3. What is your reason for keeping dogs [select all that applies]
   1. Protection (2) Companionship (3) Hunting (4) Herding (5) Eating (6) Others___
4. Where do you keep your dogs (i.e., Dog housing)

(1) Specially constructed cages (2) Anywhere on the premises

1. Where do you allow your dog to go to?

(1) Never allowed to leave the premises (2) Allowed to roam freely in the neighbourhood

1. Is/are your dog(s) vaccinated?

(1) Yes (2) No (3) Others [one or some are vaccinated]

1. If yes, do you vaccinate your dog every year?

(1) Yes (2) No

1. Ever seen a rabid animal

(1) Yes (2) No

1. Has any of your dogs been diagnosed with rabies

(1) Yes (2) No

1. If yes, what did you do?________
2. Are you vaccinated against rabies?

(1) Yes (2) No

1. Have you been bitten by a dog?

(1) Yes (2) No

1. If yes, what did you do? Select all that applies

(1) Did nothing (2) sought help from a health professional (3) I took rabies post-exposure prophylaxis (PEP) (4) Tied the dog and waited for signs of rabies (5) Washed and rinsed the site of bite (6) practiced traditional treatment (7) Other ______
